# Identification and antiviral mechanism of a novel chicken-derived interferon-related antiviral protein targeting PRDX1

**DOI:** 10.1371/journal.ppat.1013495

**Published:** 2025-09-08

**Authors:** Jing Chen, Peiheng Li, Letian Li, Ju Li, Yuhang Jiang, Wancheng Zou, Pengfei Hao, Zihan Gao, Jiayi Hao, Xiaoshuang Shi, Dongliang Fei, Mingxiao Ma, Guoqing Wang, Chang Li

**Affiliations:** 1 State Key Laboratory for Diagnosis and Treatment of Severe Zoonotic Infectious Diseases, Key Laboratory of Zoonosis Research, Ministry of Education, College of Basic Medical Science, Jilin University, Changchun, China; 2 Changchun Veterinary Research Institute, Chinese Academy of Agricultural Sciences, State Key Laboratory of Pathogen and Biosecurity, Key Laboratory of Jilin Province for Zoonosis Prevention and Control, Changchun, China; 3 College of Animal Husbandry and Veterinary Medicine, Jinzhou Medical University, Jinzhou, China; University of Tokyo Graduate School of Medicine Faculty of Medicine: Tokyo Daigaku Daigakuin Igakukei Kenkyuka Igakubu, JAPAN

## Abstract

In this study, we identified a new chicken-specific protein, named chicken interferon-related antiviral protein (chIRAP) after sequence analysis and comparison, which inhibited the proliferation of various viruses including influenza A virus (IAV) and Newcastle Disease Virus (NDV) *in vitro*, and chicken embryos with high expression of chIRAP reduced IAV infection. Mass spectrometry analysis of chIRAP interacting proteins and screening of interacting proteins affecting the function of chIRAP revealed that the deletion of endogenous chicken peroxiredoxin 1 (chPRDX1) significantly reduced the antiviral effect of chIRAP. In order to clarify the functional site of chPRDX1 affecting the antiviral effect of chIRAP, we constructed the point mutants of chPRDX1 based on the results of molecular docking (D79A, T90A, K93A, Q94A, R110A, R123A), and screened the sites affecting the antiviral effects of chIRAP by knockdown of endogenous chPRDX1 combined with the overexpression mutant strategy, the results showed that the mutations in the sites affected the antiviral effects of chIRAP to different degrees, with D79A being the most significant, and the D79A mutation of chPRDX1 reduces the ability of chPRDX1 to regulate reactive oxygen species (ROS). chIRAP may exert antiviral effects by regulating the intracellular ROS balance at the D79 site of chPRDX1. In conclusion, we identified a novel chicken-derived antiviral protein, clarified its antiviral effects and preliminarily explored its mechanism of action, which provides a new tool and option for the prevention and treatment of avian-origin viral diseases, especially avian-origin related zoonotic diseases.

## Author summary

We discovered a novel chicken-derived broad-spectrum antiviral protein, chicken interferon-related antiviral protein (chIRAP), which inhibited the proliferation of various viruses including influenza A virus (IAV) and Newcastle Disease Virus (NDV) *in vitro*, and chicken embryos with high expression of chIRAP reduced IAV infection. Mass spectrometry analysis of chIRAP interacting proteins and screening of interacting proteins affecting the function of chIRAP revealed that the deletion of endogenous chPRDX1 significantly reduced the antiviral effect of chIRAP. Different site mutations D79A, T90A, K93A, Q94A, R110A, R123A of chPRDX1 have different effects on the antiviral effect of chIRAP, but D79 has the most significant effect. chIRAP may exert its effects through the D79 site of PRDX1. This will provide a new means and research direction for the prevention and control of avian-origin viral or zoonotic viral diseases, and potential targets for the development of antiviral drugs for other species.

## Introduction

Infectious diseases, especially zoonosis, pose unprecedented challenges to public health security and property economy, and the development of novel prophylactic and therapeutic drugs and vaccines is a priority response for prevention and control [1,2]. Currently, vaccines remain one of the best strategies for inducing robust humoral and cellular immunity against human diseases. However, for viruses that mutate and spread rapidly, numerous factors such as viral immune escape, poor antibody cross-protection, and individual differences can reduce vaccine protection. At the same time, vaccine hesitancy, a product of the game between the public, the government and the scientific community, has become a major obstacle to the construction of the “immune barrier”, which cannot be ignored in almost any policy for the prevention and control of infectious diseases. Therefore, along with the development of fully functional vaccines, the development of safe, effective and broad-spectrum antiviral drugs should also be focused on.

The innate immune response is the first line of defense against viral infection. Pattern recognition receptors (PRRs) in host cells sense molecular patterns associated with pathogens and trigger a series of signalling cascades and subsequent gene activation, with the response of the Interferon (IFN) pathway being particularly important [3]. IFN can induce a variety of IFN-stimulated genes (ISGs), such as interferon-induced transmembrane (IFITMs), interferon-stimulated gene 15 (ISG15), myxovirus-resistant 1 (Mx1), protein kinase R (PKR), etc [4]. Chickens lack retinoic acid-inducible gene 1 (RIG-I) and interferon regulatory factor-3 (IRF3), depend on melanoma differentiation-associated protein 5 (MDA5) and laboratory of genomics and physiology 2 (LGP2) to activate IFN-β, and mediate the stimulator of interferon genes (STING) and mitochondrial antiviral signaling protein (MAVS) pathways through interferon regulatory factor-7 (IRF7) against DNA and RNA viruses [5,6], goose IFN-α and IFN-γ significantly inhibit Duck Plague Virus (DPV) replication through positive feedback regulation by inducing myxovirus resistance protein (Mx) and oligoadenylate synthetase-like (OASL) expression [7]. Mx proteins are interferon-induced GTPases that act by targeting viral capsids or inhibiting viral replication. The antiviral activity of chicken Mx proteins is influenced by amino acid position 631, with the asparagine (Asn) type having antiviral activity and the serine (Ser) type being inactive [8,9]. Duck Mx protein significantly inhibited RNA viruses such as vesicular stomatitis virus (VSV) and Newcastle disease virus (NDV) *in vitro*, and its antiviral mechanism was related to viral capsid binding and GTPase activity. Goose Mx protein inhibits Tambusi virus (TMUV) replication by capturing viral particles induced by interferon, showing cross-species antiviral potential [10]. The OAS family includes OAS1 and OASL, with OASL being more critical in avian species. Goose OASL inhibits TMUV by a non-enzymatic mechanism, and its antiviral activity does not depend on a conserved enzymatic active site or ubiquitin-like structural domain (UBL) but rather acts through direct interaction with viral nucleic acids or by mimicking ISG15 function. Chicken OASL complements mammalian ISG15 function by interacting with host proteins through the LRLRGG motif and enhancing RIG-I-mediated IFN induction. Ostriches are the only avian species to possess both OAS1 and OASL, and the enzymatic activity of OAS1 may play a complementary role in specific viral infections [10,11]. Protein Kinase R (PKR) inhibits viral protein synthesis by phosphorylating eukaryotic translation initiation factor eIF2α. Activation of chicken PKR in response to dsRNA stimulation significantly inhibits the replication of a variety of viruses, but its specific role in avian IAV infection remains to be validated [12]. IFITMs are small-molecule transmembrane proteins, which have become the focus of antiviral research in recent years due to their broad-spectrum antiviral activity and unique ability to inhibit viral invasion. Since IFITMs were first reported to have antiviral activity in 1996 [13], they have now been shown to restrict a wide range of viral infections, including IAV, Ebola virus (EBOV), Severe Acute Respiratory Syndrome virus (SARS-CoV-1/2), Human Immunodeficiency Virus (HIV), and Zika virus, which are some of the viruses that are seriously endangering human health and social stability [14].

IFITM proteins are explicit antiviral effectors, which can inhibit the IFITMs proteins are well-defined antiviral effectors that are multifunctional limiting factors capable of inhibiting viral infection. Although our understanding of them, based on current findings, is that they inhibit viral entry through their effects on cell membranes, e.g., [15], they can restrict viral entry by disrupting membrane properties, there are still many unknowns and unanswered questions about their underlying cellular biology considering other antiviral mechanisms that have been described, e.g., [16], IFITMs proteins are differentially associated with viral translation, vesicular transport, nuclear translocation and protein hydrolysis processing. In addition to this, functions involving lipid raft composition [17], receptor signaling [18], vesicular transport [19] and immune regulation [20] have also been proposed. Therefore, it is of great significance to deeply study and explore the roles and mechanisms of IFITMs and explore other physiological roles. While studying the antiviral effects of chicken IFITMs, we identified an open reading frame (ORF) located within the chIFITM2 motif with *in vitro* antiviral effects, named chicken interferon-related antiviral protein (chIRAP), which inhibited the proliferation of various viruses including IAV and NDV *in vitro*, and chicken embryos with high expression of chIRAP reduced IAV infection. Affinity proteomics analysis of chIRAP interacting proteins and screening of interacting proteins affecting the function of chIRAP revealed that the deletion of endogenous chicken peroxide-reducing protein family 1 (chPRDX1) significantly reduced the antiviral effect of chIRAP.

The peroxide-reducing protein family (PRDX) consists of sulfhydryl proteins that function as antioxidants. PRDX proteins catalyze the reduction of different peroxide substrates and are essential for H_2_O_2_-mediated cellular signaling [21*–*23]. There are six isoforms of PRDX, which are classified into three subclasses based on the number and position of cysteine residues, namely, subclass 2-Cys (PRDX1–4), atypical 2-Cys (PRDX5), and 1-Cys (PRDX6) [24,25]. In addition to acting as an antioxidant, PRDX proteins are involved in a variety of physiological processes, including cell growth, differentiation, apoptosis, immune response, and metabolism, as well as intracellular Reactive oxygen species (ROS) homeostasis [26*–*29].

PRDX uses thioredoxin as an intermediate electron donor to reduce ROS and exerts antioxidant effects mainly through the conversion of H_2_O_2_ to H_2_O and O_2_, thereby protecting cells from H_2_O_2_-induced apoptosis. Related studies have been conducted in a variety of solid tumors, such as breast cancer, oral tumors, hepatocellular carcinoma and colorectal cancer [30*–*37]. In antiviral studies, PRDX1 and PRDX4 protect against oxidative damage caused by Respiratory Syncytial Virus (RSV) infection [38], however, it has also been shown that knockdown of PRDX1 inhibits replication of IAV (A/WSN/33) [39]. Therefore, what role PRDXs plays in viral infections still need to be further explored. In this study, mutations in the D79, T90, K93, Q94, R110, and R123 loci of PRDX1 affected the antiviral effects of chIRAP to different degrees, with D79 being the most significant, suggesting that chIRAP may exert its antiviral effects by acting on PRDX1.

In conclusion, we identified a novel chicken-derived antiviral protein, clarified its antiviral effects, and preliminarily explored its mechanism of action, providing a new tool and option for the prevention and treatment of avian-derived viral diseases, especially avian-related zoonoses. However, it remains unknown how chIRAP affects the function of PRDX1 and regulates downstream signaling pathways. Additionally, establishing effective animal models may be necessary for further research on the functional mechanisms.

## Results

### *chIRAP* gene discovery and sequence analysis

**Fig 1:**
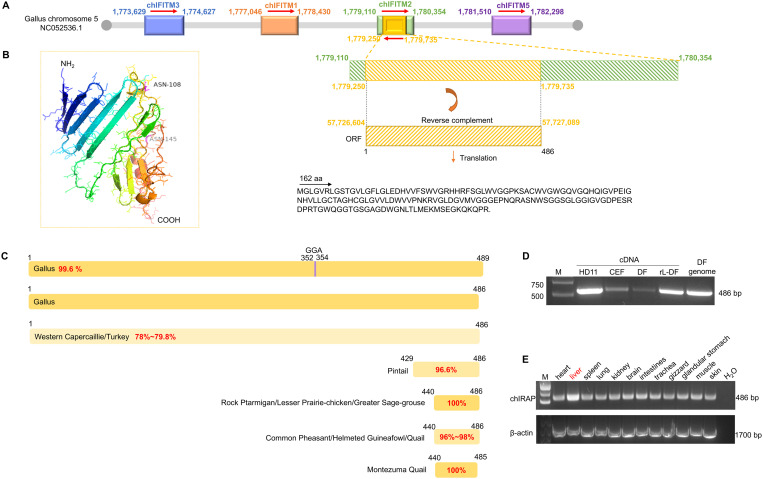
Sequence characteristics of *chIRAP.* (A) Structure of *chIRAP* CDS. *chIRAP* CDS is 486 bp located in chicken chr5 from 1,779,250-1,779,735 bp (yellow). (B) Three-dimensional prediction of *chIRAP* by alpha fold. *chIRAP* contains 10 β-folds and 4 α-helices (shadow), and exists in position 108, 145 two glycosylation sites. The results of comparative analysis of *chIRAP* and other bird nucleotide sequences are shown in S1 Table. (C) Sequence alignment of *chIRAP* and different avian genes. The target gene exists in two forms in the chicken genome: one is the reference sequence of this study and the other has a GGA mutation at positions 352-354 compared to the reference sequence. (D) PCR amplified the *chIRAP* from chicken-derived cell cDNA (HD11, CEF, DF-1) (chIRAPF/chIRAPR). PCR amplification of target fragments in cDNAs and genomes of different chicken-derived cells. (E) PCR amplification of *chIRAP* in different tissues cDNA of chicken (chIRAPF/chIRAPR). Highest levels of target genes in liver (red).

Interference induced transmembrane proteins (IFITMs) are very important class of antiviral proteins [40]. When studying chIFITMs, through sequence comparison, a 486 bp ORF was found within the motif of chIFITM2 unexpected, which attracted our interesting. The ORF has the opposite direction of transcription to that of chIFITM2, and the structure prediction showed that the sequence contains 10 β-folds and 4 α-helices, and exists in position 108, 145 two predicted glycosylation sites (Fig 1A-1B). In addition to analyzing the transcribed region of the sequence, about 3.0 kb of upstream sequence and about 2.0 kb of downstream sequence were analyzed, and two possible promoter core regions were found in the upstream (underlined promoter region, TATA box in green, and transcriptional start site in red), and transcription factor binding sites (bolded, underlined) was predicted for the promoter region, while GAAANN elements (pink) were identified near the translational start site, which were reported to be essential for virus-induced type I IFN-mediated binding of IRF family members (S1A Fig). Based on the properties, we named it chicken interferon-related antiviral protein (chIRAP).

Then, we compared the sequence in different avian genes, and found that there are two forms of the gene in chicken, one is 486 bp, which is a complete ORF, and the other is three more nucleotides (GAA) at 352–354, and the second and 64th positions are not G, but A. In other avian genes, we found that there is a sequence with 78% homology to the target gene on grouse chromosome 6 (OX596300.1), but there is a stop codon in the centre which cannot be translated correctly. The region between 400 and 486 in the target sequence is a shared sequence among certain bird species (Quail, Rock Ptarmigan, Lesser Prairie-chicken, Greater Sage-grouse, Montezuma Quail, Common Pheasant, Helmeted Guinea-fowl, Quail), suggesting that this region may be a functional region (Fig 1C, S1 Table).chIRAP has not been observed in other species.This result shows that the target gene is also present in other avian species besides chicken. However, it is not completely consistent, which may be a difference formed during genetic evolution, or the result of selection.

Next, we verified the presence of the gene in cDNA derived from different avian cells and tissues, and the results showed that the target fragment was amplified in both cells and tissues, and the highest abundance was found in the liver among different tissue samples from chickens (Fig 1D-1E). Further analysis of the protein properties showed that the protein consisted of 161 amino acids, with a theoretical pI of 7.82, a relative molecular weight of 16.7 ku, half-life in yeast and *E. coli* were both greater than 20 h and both greater than 10 h, respectively. While the half-life in mammalian cells was about 30 h, with an instability index of 34.26, which made it a stable protein (https://web.expasy.org/cgi-bin/protparam/protparam) with no transmembrane region (https://services.healthtech.dtu.dk/services/TMHMM-2.0/).

### Viral infection and interferon stimulation upregulate chIRAP expression

**Fig 2:**
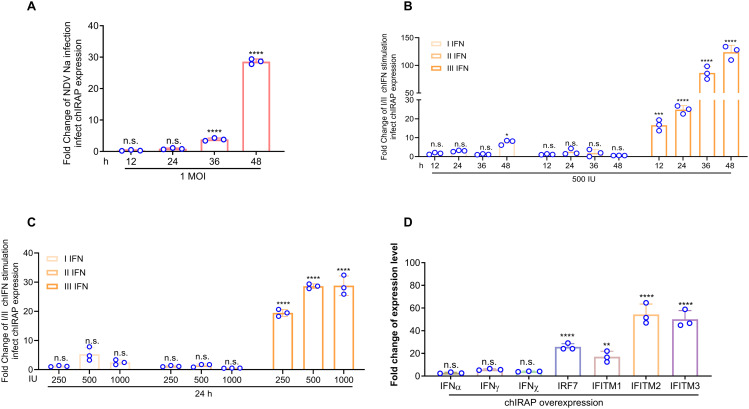
Viral infection and interferon stimulation upregulate *chIRAP* expression. (A) qRT-PCR results showed that viral infection (NDVNa-EGFP, 1 MOI) promoted *chIRA*P expression at 12-48 h (qchIRAPF/qchIRAPR). (B-C) qRT-PCR analysis of the effect of IFN stimulation on *chIRAP* transcript levels. (B) I/II/III chIFN (chIFNβ, chIFNγ, chIFNλ3, 500 IU) stimulation up-regulated *chIRAP* expression in a time-dependent; (C) I/II/III chIFN (chIFNβ, chIFNγ, chIFNλ3, 250,500,1000 IU) stimulation up-regulated *chIRAP* expression in a dose-dependent manner (24 h). The primers used are shown in S1 Table (qchIRAPF/qchIRAPR). (D) Transcript levels of interferon pathway-related genes were analysed by qRT-PCR in DF-1 cells after overexpression of *chIRAP*. Fold change is the multiple of the background expression level of the target gene in blank cells.

Therefore, we examined whether *chIRAP* could respond to viral infection and interferon stimulation. Viral infection up-regulated the expression of *chIRAP* compared with the mock group (Figs 2A, S1B). Type I IFN and chicken Interferon lambda 3 (chIFNλ3) stimulation could induce the expression of *chIRAP* and its expression level was dose-dependent and time-dependent but not type II IFN (Fig 2B-2C). Next, the effect of *chIRAP* overexpression on the expression level of molecules related to the IFN pathway was detected (Fig 2D), and IRF7, IFITM1, IFITM2, IFITM3 mRNA expression levels were significantly increased, suggesting that *chIRAP* is closely related to the interferon pathway and may have antiviral effects.

### Analysis of antiviral effects of chIRAP

**Fig 3:**
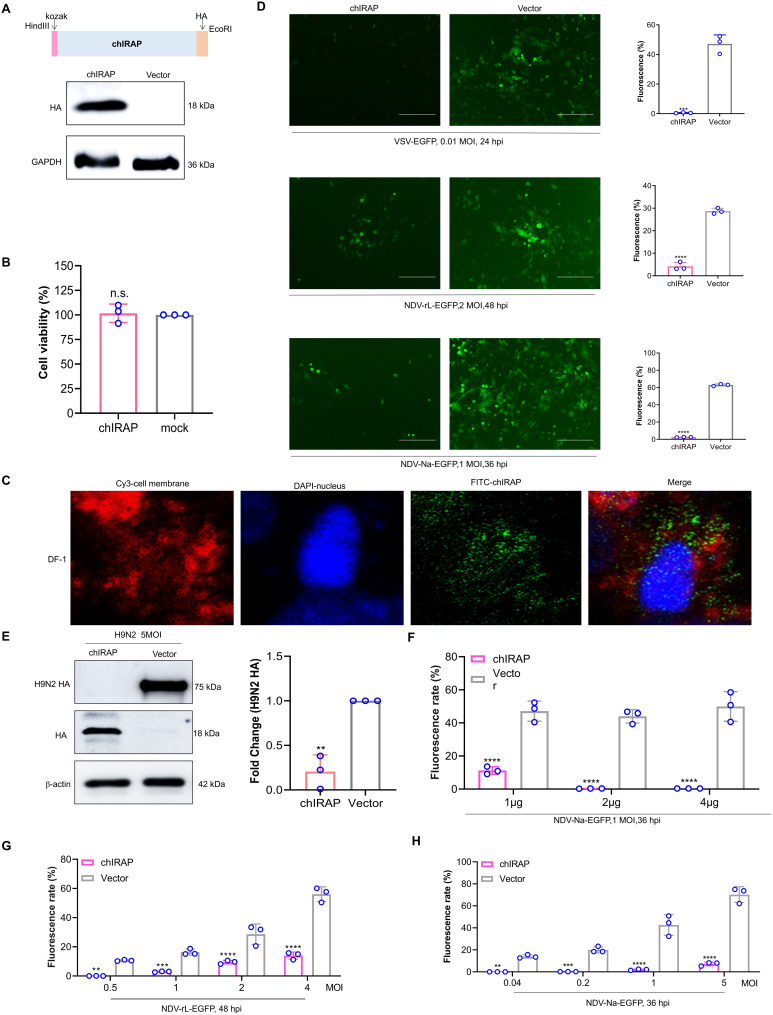
Analysis of antiviral effects of chIRAP. (A) Schematic diagram of chIRAP recombinant expression plasmid construction. Expression of chIRAP identified by WB. (B) CCK8 analyses the effect of chIRAP overexpression on cell viability. (C) Cell localization of chIRAP was analyzed by laser confocal analysis. (D) Analysis of the antiviral activity of chIRAP by fluorescence observation and flow cytometry using 12 wells plate at 24 h after VSV-EGFP infection (0.01 MOI), 48 h after NDV-rL-EGFP infection (2 MOI), and 24 h after NDV-Na-EGFP infection (1 MOI). The scale bar corresponds to 150 mm. *, P < 0.05; **, P < 0.01; ***, P < 0.001; ***, P < 0.0001. (E) Analysis of the antiviral activity of chIRAP by WB at 48 h after Influenza A virus strains H9N2 infection. Analysis of the level of H9N2 HA gene by qRT-PCR at 48 h after Influenza A virus strains H9N2 infection (qH9N2 HAF/qH9N2 HAR). (F) The antiviral effect of chIRAP is dose-dependent. (G-H) chIRAP can inhibit viral infections with different multiplicity of infections. (G) NDV-rL-EGFP and (H) NDV-Na-EGFP. mock: Untreated blank cell control group; Vector: Transient overexpression of pcDNA3.1(+) empty vector control group.

To verify whether chIRAP has antiviral effects *in vitro*, a eukaryotic recombinant expression plasmid concluding *chIRAP* was constructed and transiently transfected into DF-1 cells (chicken fibroblast) (Figs 3A, S1C), and the results of CCK8 showed that overexpression of the target gene had no effect on the cellular activity (Fig 3B). The confocal laser results showed that chIRAP was located in the cytoplasm (Fig 3C). chIRAP overexpression significantly inhibited the proliferation of VSV-EGFP, NDV-rL-EGFP and NDV-Na-EGFP, and reduced viral infection 87.3-fold, 6.87-fold, and 24.84-fold, respectively (Fig 3D), and inhibited the expression level of H9N2 IAV HA protein (Fig 3E), suggesting it also has an inhibitory effect on H9N2 IAV. Different transfection doses of chIRAP effectively inhibit the proliferation of NDV-Na-EGFP. The antiviral effect of the high-dose group is better than that of the low-dose group, and the antiviral effect of chIRAP is dose-dependent, and a good effect is achieved at 2 μg (Fig 3F). Transient transfection of 2 μg of chIRAP has antiviral effects on different infection pluralms of different NDV strains (Fig 3G-3H). chIRAP has antiviral effects in both CEK and CEF of chicken-derived cells (S1D Fig). In the results of Fig 1C, structural domains 440–486 are conserved and may be potential functional structural domains.To analyze whether this part might be an antiviral functional structural domain, we constructed the recombinant expression plasmid of the 400–486 parts and named it ∆chIRAP. This structure can effectively inhibit viral proliferation, but its antiviral effect is slightly lower than that of the full-length of chIRAP, which suggests that the structural domain of 400–486 has an antiviral effect, but there are other structures and sites in the full-length of chIRAP that play an antiviral role (S1E Fig).

### siRNA knockdown of chIRAP increases viral infection but have no effect on the antiviral effects of chIFNλ3

**Fig 4:**
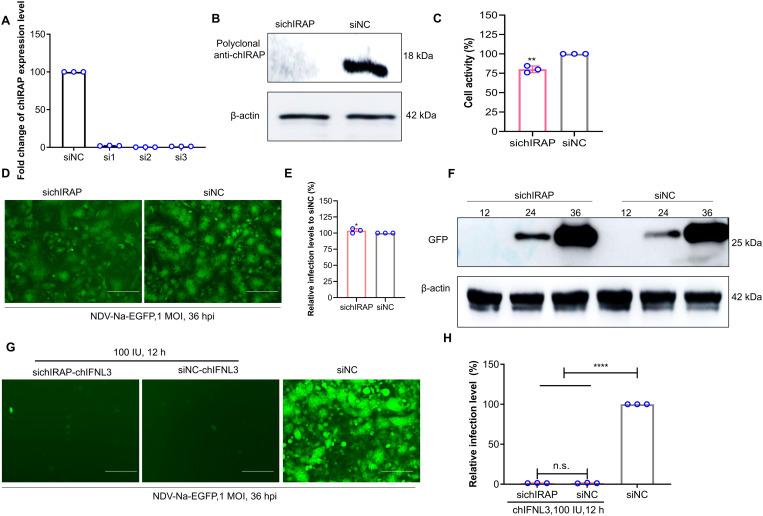
Endogenous chIRAP -deficient cells are susceptible to viral infection. (A) qRT-PCR screening for optimal siRNAs. The best interference is shown in red. (B) WB detection of siRNA interference effects. Polyclonal anti-chIRAP were used as primary antibodies and HRP labeled Goat anti-mouse IgG (H + L) as the secondary antibody. (C) CCK8 detects the effect of endogenous chIRAP deletion on cellular activity. **, P < 0.01. (D-E) Effects of endogenous chIRAP deletion on cellular resistance to viral infection analyzed by fluorescence observation(D) and flow cytometry(E) (NDV-Na-EGFP, 1 MOI, 24 h). The scale bar corresponds to 150 μm. *, P < 0.05. (F) WB analysis of the effect of endogenous chIRAP deletion on cellular resistance to viral infection at different time points of viral infection (NDV-Na-EGFP, 1 MOI, 24 h). (G) Effect of knock down chIRAP on the antiviral effects of chIFNλ3 (100 IU,12 h) by fluorescence observation. The scale bar corresponds to 150 μm. (H) Effect of knock down chIRAP on the antiviral effects of chIFNλ3 (100 IU,12 h) by flow cytometry. ns, no significant difference; ****, P < 0.0001. siNC: Negative control group for instant transfection of siRNA.

The endogenous chIRAP was interfered with by siRNA, and the level of *chIRAP* was detected by qRT-PCR to screen the optimal siRNA (Fig 4A). Meanwhile, the knockdown effect of the optimal siRNA was detected by the polyclonal antibody against chIRAP prepared in our laboratory (Fig 4B). The siRNA effectively reduced chIRAP expression, which could be used for the subsequent experiments. Unexpectedly, the knockdown of chIRAP reduced the cellular activity by about 20% (Fig 4C), suggesting that it might be a protein with an important role to play. Viral infection assay showed that the deletion of endogenous chIRAP promoted viral proliferation at 24 h (Fig 4D-4F), but did not affect the antiviral effect of chIFNλ3 [41], which suggests that although the expression of chIRAP is regulated by chIFNλ3, it may not be an important antiviral protein in the IFN pathway (Fig 4G-4H).

### chIRAP reduces the infection of IAV in chicken embryos

**Fig 5:**
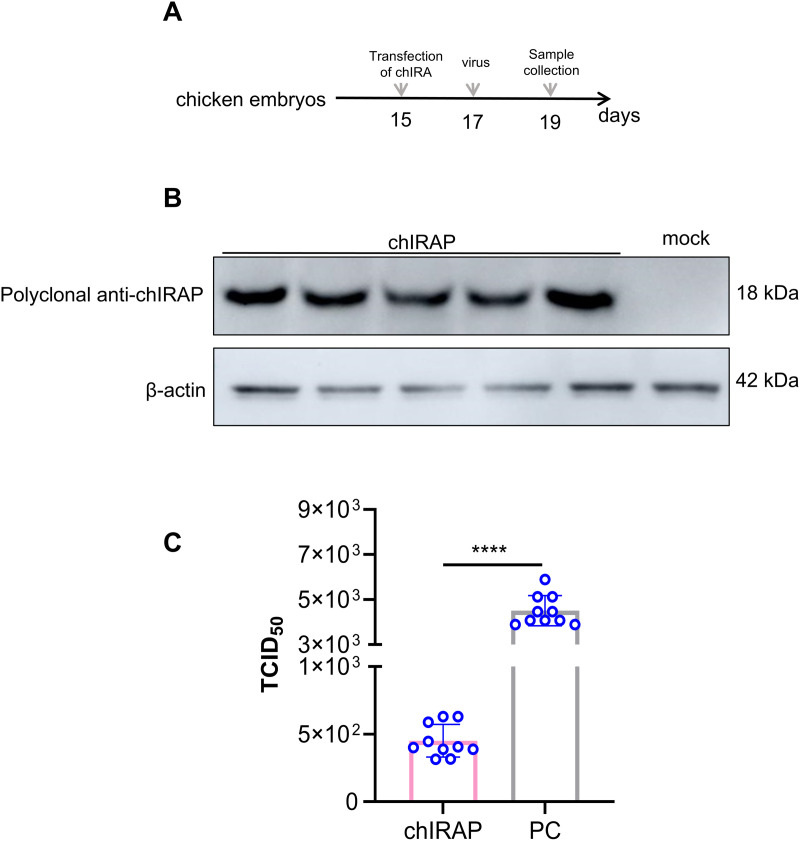
chIRAP reduces the infection of influenza virus in chicken embryos. (A) Schematic diagram of chicken embryo processing and detoxification. The 15-day-old chicken embryos were infected with the virus H1N1 (1 × 10^5^ Pfu) 48 hours after transfection with chIRAP (n = 10), and samples were collected 48 hours after the virus infection. (B) The expression of chIRAP in chicken embryos was detected by WB (Five chicken embryos randomly selected from the overexpression group were used for the detection of chIRAP overexpression). Polyclonal antibodies anti-chIRAP were used as primary antibodies and HRP labeled Goat anti-mouse IgG (H + L) as the secondary antibody. (C) Determination of viral titer in allantoic fluid. ****, P < 0.0001. mock: Untreated blank cell control group; PC: Virus-infected untreated chicken embryos group.

In order to verify the *in vivo* antiviral effect of chIRAP, we transfected chIRAP into chicken embryos (n = 10) and infected them with IAV H1N1 after 48 h. Urinary allantoic fluid and chicken embryos were collected after 48 h of viral infection, and viral virulence was measured in the urinary allantoic fluid, and protein was extracted from the embryos after tissue grinding to detect the expression of chIRAP (five chicken embryos from the randomly selected overexpression group were examined). chIRAP was correctly expressed, and viral virulence was significantly lower in the transfected chickens than in the control group (Fig 5A-5C).

### Construction and characterisation of inducible cell lines of chIRAP

**Fig 6:**
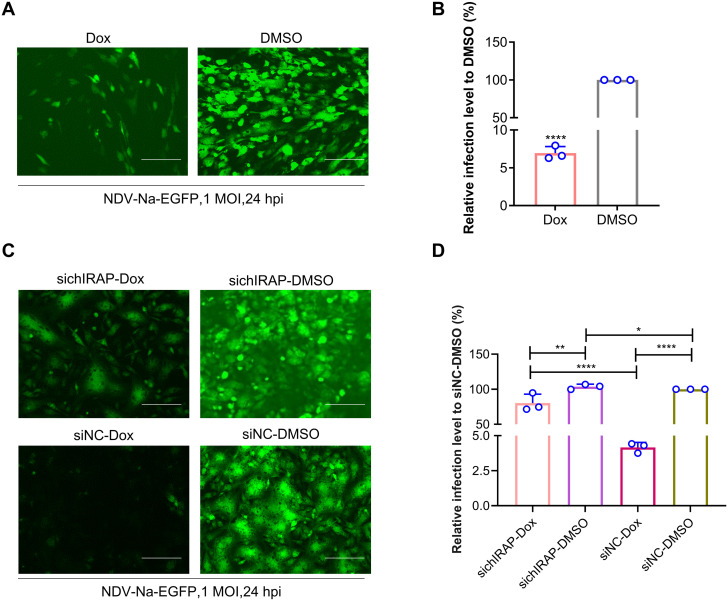
Construction and characterisation of inducible cell lines of chIRAP. (A) Fluorescence observation analysis of the antiviral effects of cell lines with optimal induction conditions (NDV-Na-EGFP, 1 MOI, 24 h). DMSO was used as a negative control. The scale bar corresponds to 150 μm. (B) Flow cytometry analysis of the antiviral effects of cell lines with optimal induction conditions (NDV-Na-EGFP, 1 MOI, 24 h). DMSO was used as a negative control. ****, P < 0.0001. (C-D) Knockdown of chIRAP in overexpressing cell lines, after transfection of siRNA for 48h, cells were induced with Dox (2.5 μg for 24 hours) and infected with NDVNa-EGFP (1 MOI,24 h), fluorescence observation(C) and flow cytometry (D) were used to analyse the infection of NDVNa-EGFP.DMSO was used as a negative control. The scale bar corresponds to 150 μm. *, P < 0.05; **, P < 0.01; ****, P < 0.001.

To better study the antiviral mechanism of chIRAP, an inducible DF-1 cell line stably expressing chIRAP was constructed (S1F Fig) and screened the induction conditions with the highest expression of the target protein (S1G Fig). Under the optimal induction conditions, the transcription level of the target protein was increased by 5 × 10^5^ fold (S1H Fig), and the cell line could effectively inhibit the proliferation of NDV-Na-EGFP with an inhibition rate of about 95% (Fig 6A-6B), and the reverse complementary experiments showed that the overexpression cell line could effectively inhibit the proliferation of the virus (Fig 6C-6D), which further demonstrated that the inducible cell lines of chIRAP functioned well, and could be used for the subsequent experiments. Then we used the cell lines to detect the stage at which chIRAP affects infection. In the virus adsorption experiment, no significant difference was observed in the viral gene copy number between the chIRAP overexpression group and the control group, indicating that chIRAP overexpression does not affect the virus adsorption process. However, in the virus entry experiment, the viral gene copy number in the chIRAP overexpression group was significantly reduced compared to that in the control group, suggesting that the chIRAP overexpression affects the entry of the virus (S2A-S2B Fig).

### chIRAP may exert its antiviral effect by interacting with PRDX1

**Fig 7:**
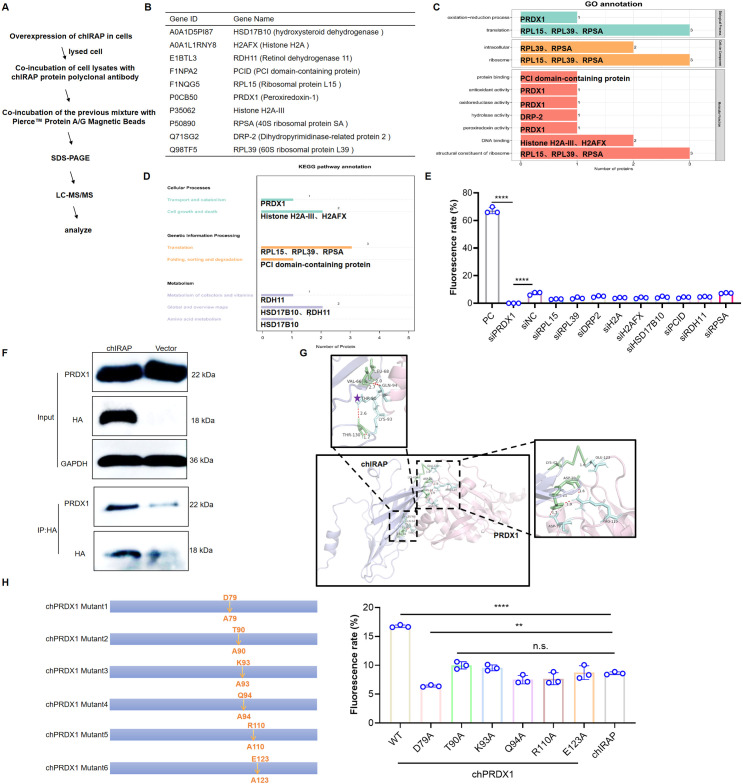
chIRAP may exert its antiviral effect by interacting with chPRDX1. (A) Label-free for analyzing chIRAP interacting proteins. (B) Screened for highly expressed proteins. (C) GO analysis of up-regulated proteins. (D) KEGG analysis of the relevant pathways involved in the upregulation of proteins. (E) Flow cytometry was used to analyze the effects of 10 proteins screened by knockdown mass spectrometry on chIRAP’s anti-NDV-Na-EGFP. (NDV-Na-EGFP, 1 MOI, 24 h). ****, P < 0.0001. siRNAs are shown in S2 Table. PC: Virus-infected untreated cell group. (F) Co-IP was used to analyze the interaction between chIRAP and PRDX1, and PRDX1 specific antibody was used to coat immunomagnetic beads.Vector: Transient overexpression of pcDNA3.1(+) empty vector control group. (G) Molecular docking predicts potential interaction sites between chIRAP and PRDX1. chIRAP forms stable hydrogen bonds with the D79, T90, K93, Q94, R110 and R123 sites of PRDX1. (H) Flow cytometry was used to analyze the PRDX1 site that affects the effect of chIRAP (NDV-Na-EGFP, 1 MOI, 24 h). ****, P < 0.0001.

**Fig 8:**
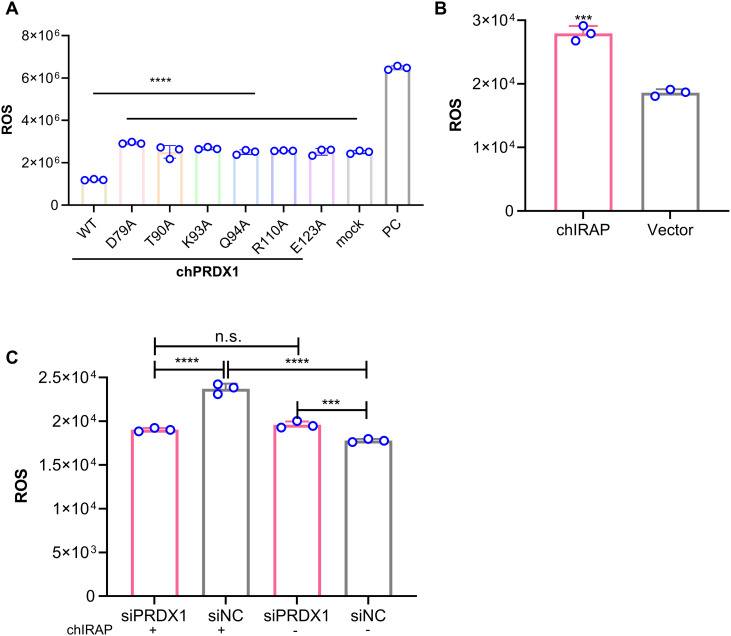
chIRAP may affect intracellular ROS balance by regulating the PRDX1 D79 site. (A) The determination of ROS levels analyzed the effect of site mutations on the ability of PRDX1 to clear ROS. mock: Untreated blank cell control group; PC: ROS-positive stimulant (Rosup) treated cell group. (B) Overexpression of chIRAP upregulates ROS levels. Vector: Transient overexpression of pcDNA3.1(+) empty vector control group. (C) Effect of endogenous PRDX1 deletion on chIRAP regulation of ROS levels. ns, no significant difference. ns, no significant difference; *, P < 0.05; **, P < 0.01; ***, P < 0.001; ****, P < 0.0001. siNC: Negative control group for instant transfection of siRNA.

Although we proved that chIRAP can be produced by viral stimulation and IFN induction and has antiviral effects, the nature and mechanism of action of this protein are still unclear. Therefore, Co-IP experiment was conducted to retrieve chIRAP interacting proteins and analysed by Lable free analysis (Fig 7A). A total of 10 highly expressed proteins were identified (Fig 7B). Next, we analyzed the pathways enriched by the above 10 proteins (Fig 7C) and found that the PRDX1 protein exists in a variety of pathways such as oxidative phosphorylation, metabolism-related, etc., indicating it may play an important role (Fig 7D). To determine whether chIRAP acts through the ten proteins screened, and which proteins play a major role, we designed siRNAs for the ten proteins and analyzed the effect of knockdown of these proteins on the antiviral activity of chIRAP. The knockdown of PRDX1 significantly enhanced the antiviral effect of chIRAP, suggesting the antiviral effect of chIRAP may be mediated by PRDX1. The antiviral effect of chIRAP may be mediated by PRDX1 (Fig 7E). The Co-IP results confirmed that there was a direct interaction between them, and the chIRAP overexpression did not affect the PRDX1 protein level (Fig 7F). Molecular docking showed that the D79, T90, K93, Q94, R110, R123 sites of PRDX1 may interact with chIRAP (Fig 7G). Therefore, we constructed a point mutant of PRDX1, overexpressed the PRDX1 mutant after interfering with PRDX1, and analyzed the effect of the mutant on the antiviral effect of chIRAP. The mutation at the D79A site significantly affected the antiviral action of chIRAP, suggesting that chIRAP acts by regulating the PRDX1 D79 site (Fig 7H). As mentioned earlier, the predominant function of PRDX1 is to scavenge ROS, does the regulation of PRDX1 by chIRAP affect intracellular ROS homeostasis? To answer the question, we first examined the ability of PRDX1 mutants to scavenge ROS, and all mutants lost the ability to scavenge ROS to varying degrees (Fig 8A). Combined with the results of Fig 7H, chIRAP may play a role in altering intracellular ROS homeostasis by regulating PRDX1 D79, overexpression of chIRAP upregulated the level of intracellular ROS (Fig 8B), and there was no significant effect of chIRAP on ROS regulation after knockdown of PRDX1 (Fig 8C), suggesting that chIRAP exerts its antiviral effect by regulating PRDX1 D79 to affect its ability to clear ROS and change the ROS balance.

## Discussion

In the process of life survival, the struggle with viruses never stops, the recognition and suppression of viruses by the organism, and the camouflage and escape of viruses in the face of recognition by the host immune system, which makes both sides evolve and upgrade in the process of the game, the host will form stable and effective antiviral patterns, such as the classical and effective IFN pathway, while different species will have differences in the speed and direction of evolution under different evolutionary pressures.

In this study, when we studied the family of IFITMs, we unexpectedly analyzed a segment of ORF (486 bp) located within the IFITM2 motif. The structure prediction showed that the sequence contains 10 β-folds and 4 α-helices, and existed two predicted glycosylation sites in position 108, 145. Results showed that it exhibited excellent antiviral activity against multiple viruses (VSV, NDV, IAV), with an *in vitro* inhibition rate exceeding 90% (Fig 6A-6B), effectively inhibiting IAV infection in chicken embryos (~14-fold, Fig 5C). Further research showed that chIRAP has only been identified in chickens and has not been detected in other avian species.

In domesticated poultry, chickens are raised in high-density farming environments, resulting in significantly higher viral transmission efficiency compared to wild birds. This “close contact” selective pressure has driven the evolution of more efficient rapid defense mechanisms in chickens’ innate immune systems unlike adaptive immunity, which relies on specific antibodies and has a slower onset, innate antiviral proteins can directly block infection at the early stages of viral entry, making them better suited for large-scale outbreaks; Birds serve as natural hosts or genetic recombination “hot spots” for various viruses such as IAV and corona viruses. As “intermediate hosts” for these viruses, chickens have evolved through prolonged interaction with them, gradually selecting inhibitory proteins targeting critical stages of the viral life cycle. For example, regulatory proteins targeting sialic acid receptors can reduce the adsorption efficiency of various viruses dependent on this receptor (such as NDV and IAV). This defensive strategy offers a greater evolutionary advantage under resource-limited conditions. Additionally, during the domestication of chickens, humans unconsciously retained individuals with stronger disease resistance. Chickens carrying highly efficient antiviral proteins are more likely to survive and reproduce. Over thousands of years, the expression intensity or functional efficiency of such genes has been amplified within chicken populations, resulting in more pronounced phenotypic differences compared to other avian species. This phenomenon of species-specific genes has also been observed in humans (ARHGAP11B) [42,43] and carp (FTRCA1) [44,45]. This may represent a core aspect of species evolutionary innovation or could result from survival pressures leading to the formation of new open reading frames through non-coding sequence rearrangements or reverse transcription events. However, further exploration and explanation are still needed.

In previous experiments, we confirmed the antiviral activity of chIRAP. While exploring the antiviral mechanism of chIRAP, we found that chIRAP participates in the entry process of NDV. However, since chIRAP is not a transmembrane protein, how does it inhibit viral entry? Based on the comprehensive data, we speculate that there may be two possible reasons. First, overexpression of chIRAP upregulates the transcriptional levels of chIFITMs (Fig 2F). chIRAP may act as a transcriptional regulator or co-activator, inducing the expression of IFITMs, thereby exerting an inhibitory effect on viral entry. Second, overexpression of chIRAP upregulates ROS levels (Fig 8B). Studies have shown that ROS can activate antiviral proteins such as IFITMs by activating transcription factors such as NF-κB and AP-1 [46,47]. chIRAP may exert its inhibitory effect on viral entry through this pathway. However, further research is needed to elucidate how chIRAP inhibits viral entry.

Chickens, as one of the most common domesticated birds worldwide, are constantly threatened by various highly pathogenic viruses, including NDV, IAV, and infectious bronchitis virus (IBV). These viral infections not only lead to a significant increase in mortality rates and a substantial decline in reproductive efficiency among poultry populations, causing global economic losses, but some viruses, such as highly pathogenic IAV, can also cross species barriers and potentially infect humans. The role of host antiviral proteins cannot be overlooked. Host antiviral proteins often target “common vulnerabilities shared by viruses”, influencing viral entry into cells and other life processes. There are evolutionary links between avian viruses and human viruses (e.g., IAV can acquire human-to-human transmissibility through mutations).

Research on chicken-derived host antiviral proteins may reveal common vulnerabilities in cross-species viral infections, providing references for antiviral drug development. Additionally, there is currently “vaccine hesitancy” in both the livestock industry and human medicine, with some people concerned about vaccine safety (e.g., allergic reactions), efficacy (e.g., escape of variant strains), or cost. The application of chicken-derived antiviral proteins offers a “non-vaccine control” approach, which does not require frequent vaccination but instead enhances the host’s own defensive capabilities to resist viruses. This is particularly important in scenarios where vaccine coverage is low or viral mutations occur rapidly. The regulatory role of chIRAP in the entry process of NDV suggests that its influence may extend far beyond a single virus, thereby providing a basis for broad-spectrum antiviral strategies and alleviating over-reliance on traditional vaccines.

Chicken host antiviral proteins serve as the “core weapons” of the innate immune system, enabling rapid responses to viral invasion. These proteins can activate subsequent immune reactions, initiate adaptive immunity, and establish a coordinated defense mechanism combining both innate and adaptive immunity. Various chicken antiviral proteins collectively form a “multi-layered defense network”, enabling chickens to effectively withstand complex viral threats. This not only ensures the health and survival of poultry but also enhances flock immunity, reduces disease incidence, and lowers breeding costs. Furthermore, the utilization of chicken-derived antiviral proteins can decrease dependence on antibiotics, antiviral drugs, and vaccines, thereby reducing the risk of drug residues, improving food safety, and promoting the transition of the poultry industry toward sustainability and ecological practices. Chicken antiviral proteins are not only crucial for poultry survival but also represent a key link connecting animal health, economic stability in the poultry sector, public health security, and biomedical innovation. In-depth studies on their functions and applications hold irreplaceable value in mitigating viral threats, ensuring the stability of the poultry supply chain, and safeguarding human health.

Although our research confirmed the antiviral activity of chIRAP, limitations such as the availability of suitable animal models and transgenic technologies prevented us from conducting in-depth in vivo studies to explore additional antiviral data and the underlying mechanisms. Further investigation is required to validate the broad-spectrum antiviral effects specifically in chickens and extend these findings to other poultry species such as ducks and geese. Moreover, broader applications in wild bird populations should be explored to comprehensively evaluate the antiviral efficacy of chIRAP and its potential for practical implementation.

In conclusion, we identified a novel chicken-derived broad-spectrum antiviral protein, chIRAP, *in vitro* antiviral experiments proved that it has a good broad-spectrum antiviral effect, which will provide a new means and research direction for the prevention and control of avian-origin viruses or zoonotic viral disease, as well as a potential target for the development of antiviral drugs for other species.

## Materials and methods

### Ethics statement

**The animal study was reviewed and approved by the experimental animal committee of Laboratory Animal Center, Changchun Institute of Veterinary Medicine, Chinese Academy of Agricultural Sciences.**

#### Cell lines.

Chicken fibroblasts (DF-1), and Baby Hamster Syrian Kidney (BHK-21) were cultured with Dulbecco’s modified Eagle’s medium (DMEM; HyClone, USA) supplemented with 10% fetal bovine serum (FBS; Gibco, USA), and 1% penicillin/streptomycin (Cytiva, USA) in a humidified atmosphere containing 5% CO_2_/95% air at 37 °C.

#### Viruses.

The velogenic Newcastle disease virus (NDV) strain Na (NDV-Na-EGFP) (GenBank No. DQ659677.1) was kindly provided by Prof. Zhuang Ding, Jilin University, China. The attenuated NDV strain (NDV-rL-EGFP) [48], H9N2 (A/Chicken/Guangdong/SS/1994; GenBank No. DQ874395.1) was kindly provided by Prof. Ming Liao, South China Agricultural University, China. NDV and IAV are produced in embryonated chicken eggs. Viral titers were determined on DF-1 cells by 50% tissue culture infective dose (TCID_50_). Vesicular stomatitis virus strains containing enhanced green fluorescent protein gene (VSV-EGFP; a gifted from Prof. ZhiGao Bu, Haerbin Veterinary Research Institute, China) [49] were determined on BHK-21 cells by TCID_50_.

#### Antibodies.

Anti-HA (5017S) was purchased from Cell Signaling Technology (Boston, USA); Anti-GFP tag Monoclonal antibody(66002–1-Ig) was purchased from Proteintech (Chicago, USA); Anti-Influenza A Virus HA (86001-RM01) was purchased from SinoBiological (Beijing, China); Anti-PRDX1(X-R0LJ39) were purchased from Abmart (Shanghai, China); Anti-β-actin (GTX109639), Anti-α-tubulin (GTX112141), Anti-GAPDH(GTX100118) were purchased from Genetex (Southern California, USA); chIRAP polyclonal antibody was prepared by our laboratory; Anti-mouse/rabbit IgG (HRP-linked antibody) (A0216/A0208) were purchased from Beyotime Biotechnology (Shanghai, China). The dilution ratio of primary antibody was 1:1000, and that of secondary antibody was 1:3000.

#### Gene amplification and bioinformatics analysis.

cDNA of *chIRAP* (GenBank No. PP558513) was generated from primary DF-1 cells and amplified by reverse transcription-polymerase chain reaction (RT-PCR) using specific primers (S2 Table).

The sequences from different species were acquired from NCBI. The phylogenetic analysis was performed using the maximum likelihood method with a bootstrap value of n = 1,000 in the MEGA program version 7. The three-dimensional structure of IFITM was determined using AlphaFold (https://alphafold.ebi.ac.uk/). Alignment analysis was performed using the DNAstar program (DNASTAR Inc., USA).

#### Plasmids construction and transfection.

All three genes were subcloned into eukaryotic expression vector pcDNA3.1 (Invitrogen, USA) with a HA tag at their C-terminal (Fig 3A). Target genes were verified through DNA sequencing (Comate Bioscience Co. Ltd., China). DF-1 cells (5 × 10^5^ cells/well) were then transfected with plasmids. At 48 h, cells were harvested. Cells were then lysed using radioimmunoprecipitation assay (RIPA) buffer (Beyotime, China) and supplemented with phenylmethanesulfonyl fluoride (PMSF, Beyotime, China).

#### CCK8 assay.

According to the manufacturer’s protocols, cell viability was assayed by the CCK8 method (Dojindo, Japan). Briefly, cells were seeded and cultured in 96/12 well plates at a density of 1 × 10^5^/5 × 10^5^cells/well in 100/500 μl of the medium for 24 h. Then treat cells as planned. At the right time, 10 μl of sterile CCK-8 solution was added to each well and incubated for another 2 h at 37 °C. The absorbance at 450 nm was determined using a microplate reader (TECAN SPAPK).

#### Virus infection.

DF-1 cells were seeded at a density of 5 × 10^5^ cells/well in 12 well plates followed by infection with indicated viruses at suitable MOIs (VSV-EGFP: 0.01 MOI; NDV-Na-EGFP: 1 MOI; NDV-rL-EGFP: 2 MOI; H9N2: 5 MOI, respectively); The vector expression protein served as a negative control.

#### Fluorescence observation.

After virus inoculation, fluorescence observation was carried out every 12 h with a Thermo Fisher Scientific EVOS M5000 microscope.

#### Western blot (WB).

Total cell extracts were prepared and separated via 10% SDA-PAGE. The proteins were transferred onto the PVDF membrane (GE Healthcare, Germany) and blocked with 5% skim milk. Membranes were incubated with specific antibodies at a room temperature of 2 h and then with HRP labeled Goat anti-rabbit/mouse IgG (H + L) at a room temperature for 1 h. The protein band was developed using the GEGEGNOME XRQ enhanced chemiluminescence (ECL) (Thermo Fisher SCIENTIFIC, USA) with the indicated antibodies.

#### qRT-PCR.

According to the manufacturer’s instructions, total RNA was extracted from cells (Sangon Biotech, China). cDNA was analyzed by quantitative PCR (qPCR) using Fast Start Universal SYBR Green Master Mix (Roche, USA) (S2 Table). Relative quantities were calculated and normalized to β-actin using the 2^-ΔΔCT^ method.

#### Construction of a cell line stably expressing chIRAP.

Common molecular cloning method was used to insert the coding sequence of target gene chIRAP into eukaryotic expression vector pLV-TRE3G to construct the pLV-chIRAP-HA plasmid with a HA tag at their C-terminal. The DF-1 cells were generated by cotransfection of the constructed plasmid and pLV-Tet3G plasmid to express the target gene. Successfully recombined monoclonal cell lines were screened by G418 and puro. qRT-PCR (S2 Table) and Western blot were used to examine the expression of the target protein in recombinant cells.

### Virus adsorption assay

Cells were infected withthe virus at 4 °C for 1 h. After this period, the supernatant was removed, and the cells were washed three times with cold PBS to remove unadsorbed virus. The cells were then harvested, and the virus adsorption levels were quantified via qRT‒PCR [50].

### Virus entry assay

The procedure for the virus entry assay was similar to that for the virus adsorption assay. After the unadsorbed virus was washed off, prewarmed medium at 37 °C was added to the cells, and the mixture was incubated for another hour to facilitate virus entry [50*–*52].

#### siRNA silencing.

DF-1 cells were seeded in 6 well plates at a density of 1 × 10^5^ cells/well overnight to reach 70–80% confluency. Then, cells were transfected with 50 nM siRNAs targeting IFITMs (RiboBio Co., Ltd., China) using Lipofectamine RNAiMAX Reagent (Thermo Fisher Scientific, USA) for 48 h according to the manufacturer’s protocol (S2 Table).

#### Flow cytometry (FCM).

EGFP-positive cells were collected at specified time points, suspended with PBS, visualized by fluorescence microscopy, and quantified by a cytoflex flow cytometer (BECKMAN COULTER).

#### Chicken embryo challenge test.

5 μg of DNA were comlexed with 0.8 μl of in vivo-jetPEI (N/P = 8) in a total volume of 10 μl. 1–2 μl were injected into the vitelline artery of chicken embryos to target the whole embryos (n = 10). Forty-eight hours later, the chicken embryos were infected with the influenza virus H1N1 (1 × 10^5^ Pfu). 48 hours after the virus infection, the chicken embryos were placed in a 4-degree environment. Six hours later, the allantoic fluid and the chicken embryos were collected, and the virus titer of the allantoic fluid was determined [53].

#### TCID_50_.

Cells were seeded into 96-well plates at 1 × 10^5^ cells per well, continuously diluting the sample 10-fold, 100 µL/well (three repetitions for each sample). After the cells had obvious CPE, the number of CPE holes under each dilution was recorded, and TCID_50_ was calculated by the Reed–Muench method.

#### Co-Immunoprecipitation (Co-IP).

For co-immunoprecipitation, the DF-1 cells were co-transfected with two expression plasmids of HA-fused protein and His-fused protein for 48 h. The cells were collected and lysed in the IP lysis buffer (Beyotime, China). Bind the antigen sample with 10 µg antibody (anti-HA or anti-His). Adjust the reaction volume to 500 µl using cell lysis buffer. Incubate the reactants at room temperature for 1–2 hours or mix overnight at 4 °C. Add the diluted sample to the tube containing pre-washed magnetic beads and gently vortex or invert to mix. Incubate the samples at room temperature with mixing for 1 hour.

Collect the beads with a magnetic stand, then remove and discard the supernatant. Add 500 µl of Binding/Wash Buffer to the tube, mix well, collect the beads with a magnetic stand and discard the supernatant. Repeat this wash twice. Add 100 µl of SDS-PAGE reducing sample buffer to the test tube and heat the sample in a heating block at 96–100 °C for 10 minutes.

#### Confocal assay.

Cells were fixed in 4% paraformaldehyde solution (Solarbio, China) for 1 hour. The cells were washed three times and closed with BSA for 1 hour. After three washes, specific primary antibodies were added and incubated at 4 °C overnight. Cells were then washed and incubated with anti-rabbit or anti-mouse fluorescent secondary antibodies for 1 hour. Images were captured by fluorescence or confocal microscopy.

#### Detection of intracellular ROS levels.

Intracellular ROS levels were detected by dihydrorhodamine 123 (MCE, USA). Cells were cultured in 6-well plates at a density of 2.0 × 10^5^ cells/well, and after overexpression of chIRAP, the cells were gently washed with PBS (Beyotime, China), followed by incubation with dihydrorhodamine 123 at 37 °C for 30 min, and then the cells were washed twice with PBS. The fluorescence of the cells was immediately measured on a flow cytometer (BECKMAN COULTER).

#### Statistical analyses.

Statistical analysis was performed using GraphPad 8.0 (GraphPad Software, SanDiego, CA) with the one-way analysis of variance (ANOVA; two-tailed, confidence intervals (CI) 95%), as indicated by the p-value. The results were statistically significant at p < 0.05. At least 3 independent experiments were evaluated for each separate set of assays. The results were expressed as the mean ± standard deviation (SD).

## Supporting information

S1 Fig (A) Structure of *chIRAP* CDS and promoter. ** **~ 5.0 Kb genomic sequence including promoter and the CDS of *chIRAP* is shown in the reverse complement order. The ORF of the *chIRAP* in yellow shadow and putative binding sites for IRF1/2, SP1, C/EBPβ, c-Jun transcription factors are bold and underlined. The TATA box is green shadow, whereas GAAANN sites are in pink shadow. The Transcription initiation site is red, and the putative polyadenylation signal is blue shadow. A predicted promoter is underlined. (B) Flow cytometry analysis of viral infection of 1MOI NDV Na-infected DF-1 cells for 12–48 h. Fluorescent green detection of viral infection in Fig 2A. ****, P < 0.0001. (C) Identification of *chIRAP* recombinant expression plasmids by double digestion. (D) chIRAP inhibits the proliferation of NDV-Na-EFDP in CEK and CEF of other chicken-derived cells. Vector: Transient overexpression of pcDNA3.1(+) empty vector control group. (E) Flow cytometry was used to analyze the anti-NDV-NA-EGFP (1 MOI, 24 h) effect of the common sequence 400–486 (∆chIRAP) in different birds. (F) Dual-enzyme identification of recombinant expression plasmids for cell line construction. (G) WB detection of target protein expression under different induction conditions. The optimal induction condition was 2.5 μg for 24 hours (red). (H) Analysis of the level of *chIRAP* gene by qRT-PCR at optimal induction conditions (qchIRAPF/qchIRAPR). DMSO was used as a negative control. ***, P < 0.001.
(TIF)

S2 Fig (A) The overexpression of chIRAP affects the virus entry process but does not affect the virus adsorption process. (B) Identification of interference effects of siPRDX1. siRNA-02 was selected for subsequent knockdown experiments Identification of chIRAP recombinant expression plasmids by double digestion. siNC: Negative control group for instant transfection of siRNA.
(TIF)

S3 Fig (A) The overexpression of PRDX1 reduces the intracellular ROS level. The PC group was the control group treated with the ROS-positive stimulant Rosup. chIRAP reduces mitochondrial membrane potential. mock: Untreated blank cell control group; PC: Virus-infected untreated cell group. (B) JC-1(Beyotime, C2003S) was used to detect the changes in mitochondrial membrane potential of cells after chIRAP overexpression. In the mitochondria of normal cells, JC-1 exists in the form of a polymer, showing bright red fluorescence and very weak green fluorescence. After overexpression of chIRAP, the intensity of red fluorescence in mitochondria decreased significantly, while the green fluorescence increased significantly. This suggests that chIRAP causes a decrease in mitochondrial membrane potential, and JC-1 cannot exist in the mitochondrial inner membrane in the form of a polymer.
(TIF)

S1 Table Sequence alignment of *chIRAP* and different avian genes. (PDF)

S2 Table Primers and siRNAs in this study. (DOC)
