# Supplementary figures and images for: Identification and antiviral mechanism of a novel chicken-derived interferon-related antiviral protein targeting PRDX1
Source: PLoS Pathog. 2025 Sep 8;21(9):e1013495. doi: 10.1371/journal.ppat.1013495 (PMC12431653; doi:10.1371/journal.ppat.1013495)

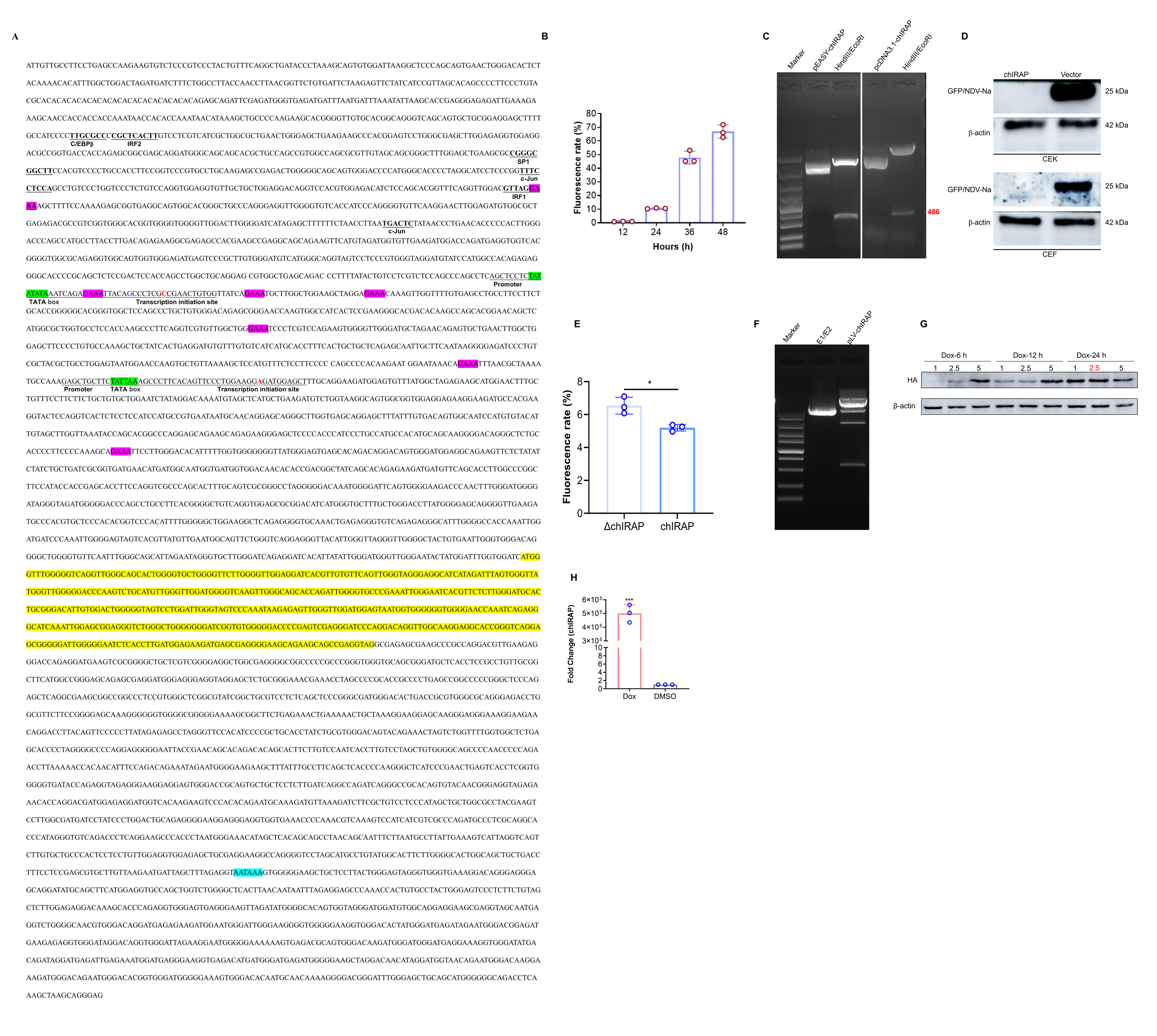

Supplement: S1 Fig — ~ 5.0 Kb genomic sequence including promoter and the CDS of chIRAP is shown in the reverse complement order. The ORF of the chIRAP in yellow shadow and putative binding sites for IRF1/2, SP1, C/EBPβ, c-Jun transcription factors are bold and underlined. The TATA box is green shadow, whereas GAAANN sites are in pink shadow. The Transcription initiation site is red, and the putative polyadenylation signal is blue shadow. A predicted promoter is underlined. (B) Flow cytometry analysis of viral infection of 1MOI NDV Na-infected DF-1 cells for 12–48 h. Fluorescent green detection of viral infection in Fig 2A. ****, P < 0.0001. (C) Identification of chIRAP recombinant expression plasmids by double digestion. (D) chIRAP inhibits the proliferation of NDV-Na-EFDP in CEK and CEF of other chicken-derived cells. Vector: Transient overexpression of pcDNA3.1(+) empty vector control group. (E) Flow cytometry was used to analyze the anti-NDV-NA-EGFP (1 MOI, 24 h) effect of the common sequence 400–486 (∆chIRAP) in different birds. (F) Dual-enzyme identification of recombinant expression plasmids for cell line construction. (G) WB detection of target protein expression under different induction conditions. The optimal induction condition was 2.5 μg for 24 hours (red). (H) Analysis of the level of chIRAP gene by qRT-PCR at optimal induction conditions (qchIRAPF/qchIRAPR). DMSO was used as a negative control. ***, P < 0.001. (TIF) [file ppat.1013495.s001.tif]

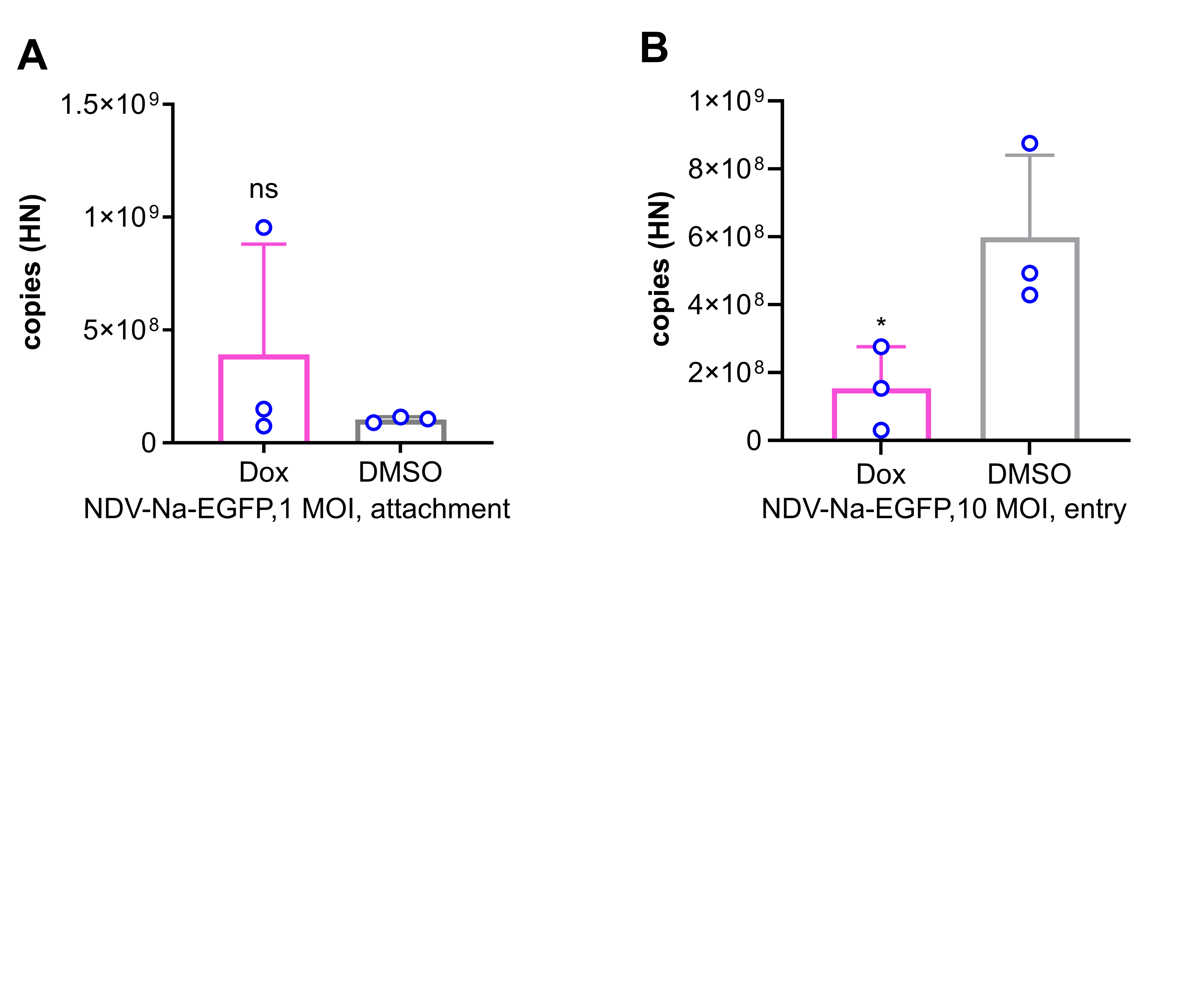

Supplement: S2 Fig — (B) Identification of interference effects of siPRDX1. siRNA-02 was selected for subsequent knockdown experiments Identification of chIRAP recombinant expression plasmids by double digestion. siNC: Negative control group for instant transfection of siRNA. (TIF) [file ppat.1013495.s002.tif]

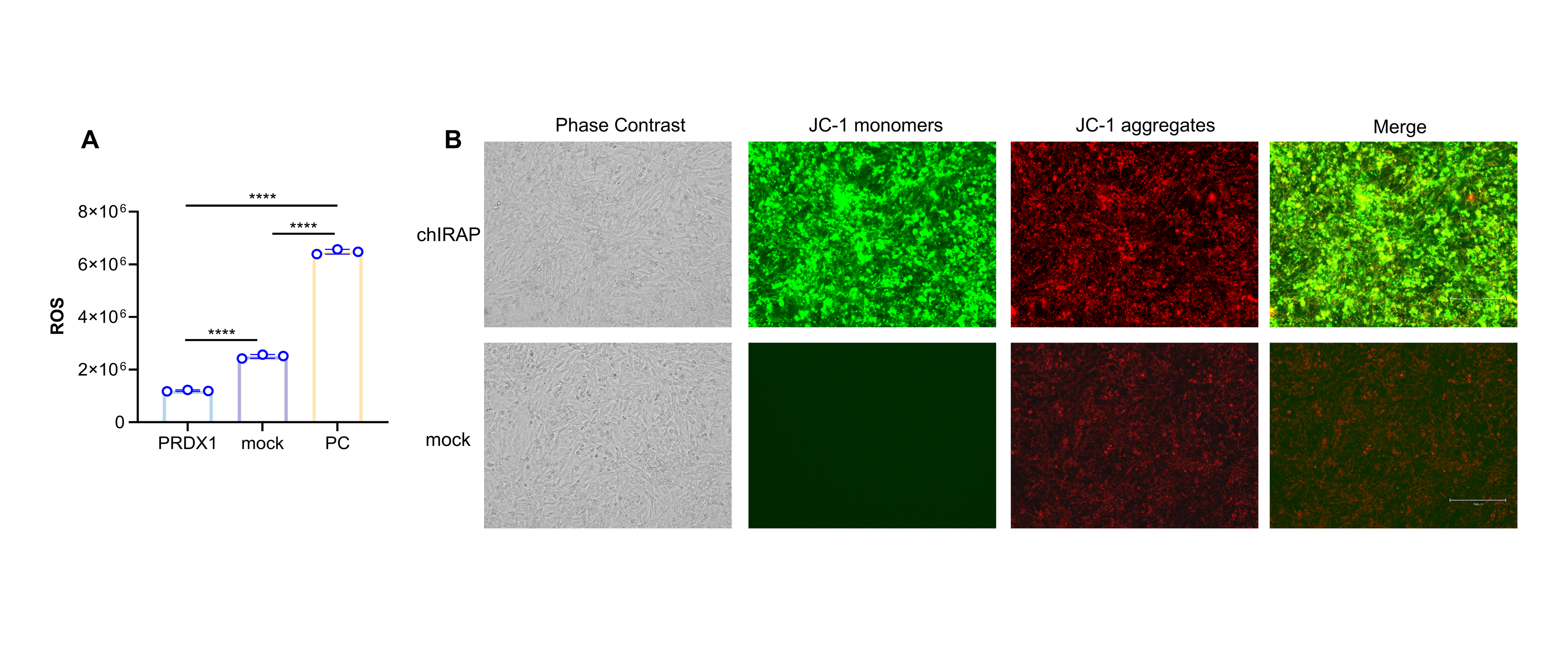

Supplement: S3 Fig — The PC group was the control group treated with the ROS-positive stimulant Rosup. chIRAP reduces mitochondrial membrane potential. mock: Untreated blank cell control group; PC: Virus-infected untreated cell group. (B) JC-1(Beyotime, C2003S) was used to detect the changes in mitochondrial membrane potential of cells after chIRAP overexpression. In the mitochondria of normal cells, JC-1 exists in the form of a polymer, showing bright red fluorescence and very weak green fluorescence. After overexpression of chIRAP, the intensity of red fluorescence in mitochondria decreased significantly, while the green fluorescence increased significantly. This suggests that chIRAP causes a decrease in mitochondrial membrane potential, and JC-1 cannot exist in the mitochondrial inner membrane in the form of a polymer. (TIF) [file ppat.1013495.s003.tif]
