# Supplementary material for: Identification and antiviral mechanism of a novel chicken-derived interferon-related antiviral protein targeting PRDX1
Source: PLoS Pathog. 2025 Sep 8;21(9):e1013495. doi: 10.1371/journal.ppat.1013495 (PMC12431653; doi:10.1371/journal.ppat.1013495)
Supplement: S1 Table — (PDF) [file ppat.1013495.s004.pdf]

Supplementary Table 1. Sequence alignment of chicken and different avian genes

Number	Species	Common name	NCBI accessions	Start	End	Fragment length	Homology (%)	Descriptions
1	Gallus	Chicken	CM008832.1	1225396	1224908	489	99.6	Three more nucleotides GGA out of 352-354 compared to the target fragment and the presence of the second, sixty-fourth position is different (G-A)
2	Gallus	Chicken	NC052536.1	1779250	1779735	486	100	Same
3	Tetrao urogallus	Western Capercaillie	OX596300.1	4860426	48690907	516	78	1-486
4	Meleagris gallopavo	Turkey	HG999685.4	4693101	4693635	535	79.8	1-486
5	Anas acuta	Pintail	OZ009967.1	31676126	31676183	58	96.6	429-486
6	Lagopus muta	Rock Ptarmigan	XM048948910.1	396	350	47	100	440-486
7	Tympanuchus pallidicinctus	Lesser Prairie-chicken	XM052693974.1	341	295	47	100	440-486
8	Centrocercus urophasianus	Greater Sage-grouse	XM042837259.1	261	215	47	100	440-486
9	Cyrtonyx montezumae	Montezuma Quail	XM065755391.1	292	247	46	100	440-485
10	Phasianus colchicus	Common Pheasant	XM031607375.1	379	333	47	98	440-486
11	Numida meleagris	Helmeted Guineafowl	XM021402690.1	337	291	46	98	440-486
12	Coturnix japonica	Quail	XM015863378.1	337	291	45	96	440-486
13	Junco hyemalis	Dark-eyed junco	CM042579.1	/	/	/	/	/
14	Malurus cyaneus	Superb Fairy-wren	CM019218.1	/	/	/	/	/
15	Columba livia	Pigeon	CM007529.1	/	/	/	/	/
16	Agelaius phoeniceus	Red-winged Blackbird	CM036718.1	/	/	/	/	/
17	Aquila chrysaetos	Golden eagle	LR606185.1	/	/	/	/	/
18	Lichenostomus cassidix	Helmet honeyeater	CM039186.1	/	/	/	/	/
19	Strigops habroptila	Kakapo	CM013766.2	/	/	/	/	/
20	Falco peregrinus	Peregrine falcon	CM007509.1	/	/	/	/	/
21	Certhia americana	American Treecreeper	CM031861.1	/	/	/	/	/
22	Molothrus ater	Brown-headed Cowbird	NC050482.1	/	/	/	/	/
23	Taeniopygia guttata	Zebra finch	CM000522.1	/	/	/	/	/
24	Anas platyrhynchos domestica	Peking duck	NC051776.1	/	/	/	/	/
25	Aptenodytes forsteri	Emperor penguin	GCA000699145.1	/	/	/	/	/
26	Pygoscelis adeliae	Adelie penguin	GCA000699105.1	/	/	/	/	/
27	Nipponia nippon	Crested ibis	GCA000708225.1	/	/	/	/	/
28	Egretta garzetta	Little egret	GCA000687185.1	/	/	/	/	/
29	Calypte anna	Anna’s hummingbird	GCA000699085.1	/	/	/	/	/
30	Chaetura pelagica	Chimney swift	GCA000747805.2	/	/	/	/	/
31	Charadrius vociferus	Killdeer	GCA000708025.2	/	/	/	/	/
32	Cuculus canorus	Common cuckoo	GCA000709325.1	/	/	/	/	/
33	Ophisthocomus hoazin	Hoatzin	GCA000692075.1	/	/	/	/	/
34	Geospiza fortis	Medium ground finch	GCA000277835.1	/	/	/	/	/
35	Manacus vitellinus	Golden-collared manakin	GCA000692015.2	/	/	/	/	/
36	Melopsittacus undulatus	Budgerigar	GCA000238935.1	/	/	/	/	/
37	Picoides pubescens	Downy woodpecker	GCA000699005.1	/	/	/	/	/
38	Struthio camelus	Common ostrich	GCA000698965.1	/	/	/	/	/
39	Tinamus guttatus	White-throated tinamou	GCA000705375.2	/	/	/	/	/
40	Corvus brachyrhynchos	American crow	GCA000691975.1	/	/	/	/	/
41	Haliaeetus leucocephalus	Bbald eagle	GCA000737465.1	/	/	/	/	/
42	Antrostomus carolinensis	Chuck-Will’s Widow	GCA000700745.2	/	/	/	/	/
43	Cariama cristata	Red-Legged Seriema	GCA000690535.1	/	/	/	/	/
44	Colius striatus	Speckled mousebird	GCA000690715.1	/	/	/	/	/
45	Merops nubicus	Carmine bee eater	GCA000691845.1	/	/	/	/	/
46	Gavia stellata	Red-throated loon	GCA000690875.1	/	/	/	/	/
47	Balearica regulorum gibbericeps	Grey-crowned crane	GCA000709895.1	/	/	/	/	/
48	Apaloderma vittatum	Bar-tailed trogon	GCA000703405.1	/	/	/	/	/
49	Phalacrocorax carbo	Great cormorant	GCA000708925.1	/	/	/	/	/
50	Phaethon lepturus	White-tailed tropicbird	GCA000687285.1	/	/	/	/	/
51	Phoenicopterus ruber ruber	American flamingo	GCA000687265.1	/	/	/	/	/
52	Podiceps cristatus	Great-crested grebe	GCA000699545.1	/	/	/	/	/
53	Fulmarus glacialis	Northern fulmar	GCA000690835.1	/	/	/	/	/
54	Tyto alba	Barn owl	GCA000687205.1	/	/	/	/	/
55	Tauraco erythrolophus	Red-crested turaco	GCA000709365.1	/	/	/	/	/
56	Cathartes aura	Turkey vulture	GCA000699945.1	/	/	/	/	/
57	Eurypyga helias	Sunbittern	GCA000690775.1	/	/	/	/	/
58	Mesitornis unicolor	Brown mesite	GCA000695765.1	/	/	/	/	/
59	Leptosomus discolor	Cuckoo roller	GCA000691785.1	/	/	/	/	/
60	Chlamydotis macqueenii	MacQueen’s bustard	GCA000695195.1	/	/	/	/	/
61	Pelecanus crispus	Dalmatian pelican	GCA000687375.1	/	/	/	/	/
62	Pterocles gutturalis	Yellow-throated sandgrouse	GCA000699245.1	/	/	/	/	/
63	Acanthisitta chloris	Rifleman	GCA000695815.1	/	/	/	/	/
64	Buceros rhinoceros silvestris	Javan Rhinoceros hornbill	GCA000710305.1	/	/	/	/	/
65	Nestor notabilis	Kea	GCA000696875.1	/	/	/	/	/
66	Haliaeetus albicilla	White-tailed eagle	GCA000691405.1	/	/	/	/	/
