# Supplementary material for: Identification and antiviral mechanism of a novel chicken-derived interferon-related antiviral protein targeting PRDX1
Source: PLoS Pathog. 2025 Sep 8;21(9):e1013495. doi: 10.1371/journal.ppat.1013495 (PMC12431653; doi:10.1371/journal.ppat.1013495)
Supplement: S2 Table — (DOC) [file ppat.1013495.s005.doc]

| Class | Primer | Sequence（5’-3’） |
| --- | --- | --- |
| PCR | chIRAPF | AAGCTTGCCACCATGGGTTTGGGGGTCAGGTTGGGCA |
|  | chIRAPR | GGTACCCTAAGCGTAGTCTGGGACGTCGTATGGGTACCTCGGCTGCTTCTGCTTCCC |
|  | pLV-chIRAPF | AAGCTTGCCACCATGGGTTTGGGGGTCAGGTTGGGCA |
|  | pLV-chIRAPR | GGTACCCTAAGCGTAGTCTGGGACGTCGTATGGGTACCTCGGCTGCTTCTGCTTCCC |
| qRT-PCR | qchIRAPF | GGGACCCAAGTCTGCATGTT |
|  | qchIRAPR | CGTGATTCCCAATTTCGGGC |
|  | qIFITM1F | GCCTGGGCTTATGTGCTCTC |
|  | qIFITM1R | TGGGGGTGATACCAGAGGTAG |
|  | qIFITM2F | ATCTTCTCCATCAAGGCCCG |
|  | qIFITM2R | ACAACACACCGACGGCTATC |
|  | qIFITM3F | GTGAAGTCCAGGGATCGCAA |
|  | qIFITM3R | GGGTCCAATGAATTCGGGGT |
|  | qIFNαF | CAACGACACCATCCTGGACA |
|  | qIFNαR | ATCCGGTTGAGGAGGCTTTG |
|  | qIFNγF | CTGACAAGTCAAAGCCGCAC |
|  | qIFNγR | CTTCACGCCATCAGGAAGGT |
|  | qIFNλF | CCTGGCCTTCCTTACCCAAG |
|  | qIFNλR | CTCAGTTTCCCAGAGGGCTG |
|  | qIRF7F | GCATCCCTTGGAAGCACAAC |
|  | qIRF7R | TTCCATTTGGCCGGATCCTC |
|  | qMx1F | CTTCACGTCAATGTCCCAGC |
|  | qMx1R | GGAATCTGATTGCTCAGGCG |
|  | qH9N2 HAF | TCGCTGGTTGGTATGGGTTC |
|  | qH9N2 HAR | AGGTCCCGTTCCGAATTGTC |
|  | qβ-actinF | TCCCGGCGCCTACTACAGGATCCGGTTGCACT |
|  | qβ-actinR | ACTGCAGGACTTCCGATTTTGGGTGTCATCT |
| siRNAs | siPRDX1 | GGAUCAACACUCCUAAGAA |
|  | siRPL15 | AGCAAGGUUAUGUUAUCUA |
|  | siRPL39 | ACAAGACCUUCAAGAUCAA |
|  | siDRP2 | CGAUCGUCUUCUGAUCAAA |
|  | siH2A | GCAACGACGAGGAGCUCAA |
|  | siH2AFX | GCAACGACGAGGAGCUCAA |
|  | siHSD17B10 | CGUGUGUGUUCAUCAGGUA |
|  | siPCID | CGCUGUACUUCGACACCAA |
|  | siRDH11 | GCACUAAAGUCACAGCAAA |
|  | siRPSA | CAGUAUAUCUACAAGAGAA |

**Supplementary Table 2. Primers in this study**
